# An exploration of teacher and student perceptions of blended learning in four secondary mathematics classrooms

**DOI:** 10.1007/s13394-020-00359-2

**Published:** 2020-11-10

**Authors:** Catherine Attard, Kathryn Holmes

**Affiliations:** Centre for Educational Research, Western Sydney University, Sydney, Australia

**Keywords:** Flipped learning, Blended learning, Mathematics, Technology, Secondary, Learning management systems

## Abstract

The COVID-19 pandemic forced many teachers around the world to make a sudden switch from face-to-face to online teaching. This shift in practice has provided an opportunity to reconsider how technology use in mathematics education can be utilised to improve student engagement. In this study, we explore four case studies of Australian secondary mathematics classrooms conducted prior to the COVID-19 pandemic to examine how teachers are using blended learning approaches and how their students perceive these pedagogical practices. Findings across all four sites indicate that technology use expands student opportunities to engage with mathematics learning through the provision of multiple pathways and methods of access. Specifically, we find evidence supporting the use of blended classroom teaching strategies to provide differentiation and personalised learning approaches; visualisation and dynamic manipulation of mathematics concepts; and alternative methods for teacher-student feedback and communication. We argue that the student learning experience in mathematics can be enhanced through a variety of blended learning approaches by allowing for diverse points of access to learning opportunities which are more closely aligned to individual learning needs and free from the temporal constraints of the classroom.

## Introduction

Prior to the COVID-19 pandemic, digital technology use in mathematics classrooms was reported to be inconsistent in quality, quantity, and effectiveness (OECD 2016). Although some viewed the use of digital technology as an educational imperative (Bower 2017), there were still many questions regarding how and when it should be used, and whether its use transformed and improved student experiences of mathematics education. The educational crisis caused by COVID-19 resulted in teachers being forced to rely on digital technology as the prime teaching and learning resource regardless of their existing technology-related beliefs and practices. This forced and sudden change could be viewed as an opportunity for significant shifts to occur in how mathematics educators use technology in future face-to-face, online and blended classroom teaching.

Technology use can potentially drive disruption in mathematics education which is imperative given the international concerns about student disengagement and falling enrolments in senior mathematics courses (Thomson et al. 2017). However, for a range of reasons, teacher resistance to technologically driven innovation within classroom teaching is not uncommon. Tangney and Bray (2013) suggest that although the affordances of digital mobile technologies align with a social constructivist teaching approach that promotes collaboration, communication, creativity, and problem-solving, technology use overwhelmingly continues to be restricted to content consumption, resulting in a regression towards a traditional approach of teaching mathematics (Attard 2015; Orlando and Attard 2016).

Integrating digital technologies effectively into mathematics teaching and learning is a complex task requiring the consideration of many elements including pedagogy, content and student learning. Uses of digital technologies in mathematics can be ineffective, distracting, or even dangerous when not integrated into the learning process in meaningful ways (Attard 2015; Freeman et al. 2017). Online learning environments provide affordances that could allow mathematics teachers to redefine practices as they currently occur in mathematics classrooms, disrupting traditional methods to mediate meaningful student-student and student-teacher interactions through blended and flipped learning approaches.

In this paper, we explore the perceptions of teachers and students from a study of four Australian secondary mathematics classrooms conducted prior to the COVID-19 pandemic. Data informing this paper is drawn from a larger multiple case study of 10 Australian mathematics classrooms ranging from pre-school to Year 12 (see Attard and Holmes 2020a; Attard and Holmes 2020b). Results of the study indicated interesting commonalities across the four secondary case study classrooms relating the varied blended learning approaches and uses of learning management systems (LMS). Little research relating to the perceived influence of LMS within mathematics classrooms currently exists yet the use of such systems has now become more common as a result of the forced shift to online learning during the COVID-19 pandemic. This has made it important to understand how blended learning strategies and LMS are being utilised and how teachers and students perceive their influences on the mathematics learning experience.

For the purpose of this paper, we explore the practices of four secondary mathematics teachers concerning the strategies and effects of blended learning approaches. We investigate how their nuanced pedagogical approaches specifically influenced how mathematics learning resources were made accessible to students and, as a result, how they influenced students’ experiences of secondary mathematics education. A clearer understanding of the intricacies involved in blended learning in mathematics and the related perceptions of teachers and students will provide insights for researchers, teachers, and pre-service teacher educators as we begin to understand the evolving educational landscape post COVID-19. These insights may assist educators in developing methods that are helpful in improving students’ access to elements of mathematics education such as learning resources, regardless of device or software, and the ways they redefine learning spaces and teaching practice. Further, the insights may provide new and valuable pathways for research in mathematics education.

## Background

### Technology and engagement with mathematics

The declining number of students entering higher levels of mathematics continues to create concern internationally (Morsy et al. 2018; Thomson et al. 2017). There are multiple factors related to student underachievement and disengagement in mathematics. One reason for the decline stems from the gaps in knowledge that occur when students fail to learn or understand critical mathematical concepts (Hoyles 2016). One common factor is the fast pace of learning that is typical in secondary schools (Boaler 1997; Hallam and Ireson 2005) which can result in these gaps in conceptual understanding, leading to student disengagement and their failing to continue the study of mathematics beyond the compulsory years, potentially limiting life and career opportunities. Expectations of improved student engagement through the use of digital technologies is widely reported in literature (e.g., Beavis et al. 2015; Bray and Tangney 2015; Pierce and Ball 2009), so it is clear that we need to understand if and how the use of technology-related practices contribute to student engagement with mathematics (Attard and Holmes 2020a).

The use of and access to technology is regarded as a necessity in today’s classrooms and some countries have embedded its use in mandated curricula (For example, Australian Curriculum Assessment and Reporting Authority (ACARA) 2010). One of the most significant benefits of technology involves opportunities for teachers to personalise learning through flexible, blended formats. In a study investigating the influence of digital technologies in four primary schools, Robinson and Sebba (2010) found when students engaged with and had ownership of digital technologies the school’s capacity to provide opportunities for personalised learning increased. In an Australian study, Hilton (2018) found the use of iPads assisted in addressing a diversity of student needs through the use of apps that provided the ability for students to work at an appropriate level. Personalised learning can still occur without digital devices; however, teachers can take advantage of the affordances of technology to vary instruction and provide student-controlled learning paths. While it might be argued that student-controlled learning paths may not be conducive to learning progress if left unmonitored, many contemporary education apps also provide teachers with frequent formative assessment and progression data aligned to curriculum standards (*Maths Pathway*, *Mathletics*, *Matific*, *Prodigy*, for example). Others also offer instant feedback to students. In some apps, this then leads to tailored learning pathways that can extend learning or provide intervention (Roblyer and Hughes 2019).

Digital technologies offer other innovative ways for students to learn and engage with mathematics through their capacity to enable learning anywhere and anytime through blended approaches, as well as the ability to capture, annotate and share multimedia. There are a range of pedagogical opportunities and implications afforded by the use of digital technologies that extend beyond the nature of the tools and software apps utilised, to the learning intentions of the teacher and the ways in which students interact with the technologies and with each other (Calder et al. 2018). Each of these implications creates opportunities to influence the teaching practice in mathematics classrooms. Digital devices provide tools that are dynamic, graphical, and interactive, providing students with opportunities to “explore mathematical objects from different but interlinked perspectives, where the relationships that are key for mathematical understanding are highlighted, made more tangible and manipulable” (Hoyles and Noss 2009, p. 132). Students can make practical use of technology in mathematics for “genuine and productive purposes, rather than for the application of rote-learned formulae and procedures to contrived scenarios” as would be typical in traditional mathematics classrooms (Bray and Tangney 2017, p. 257).

Further, digital devices afford connectivity within and between classrooms and promote collaboration and collective reflection and manipulation that can be either synchronous or asynchronous (Hoyles and Noss 2009), providing the opportunity to extend learning opportunities beyond the mathematics classroom. These affordances are becoming increasingly popular in schools and have led to many schools developing policies requiring all students to have access to a digital device (Technology for Learning Program - Information Technology Directorate 2013). In turn, the increase in personal devices has resulted in opportunities to redefine learning spaces within and outside of schools, through a broad range of blended learning approaches.

### Redefining learning spaces through blended learning

A blended learning approach combines online and face-to-face teaching. Flipped learning is considered by some as a separate approach, yet others consider it as a specific model of blended learning that has a clear delineation between online and face-to-face instruction (Borba et al. 2016; Polly and Casto 2019). At its most basic, the flipped classroom approach is intended to make better use of classroom time. Rather than expose students to new materials within mathematics lessons, students are expected to access pre-prepared materials before their lessons (Gilboy et al. 2015; Lo and Hew 2017). Pre-learning occurs outside the classroom and class time is then used to maximise opportunities for teacher/student interaction, collaboration, provision of remediation, and application of the learning that occurs off-site (Bhagat et al. 2016; Weinhandl et al. 2018). The flipped learning approach includes variations that range from the simple provision of direct instruction via the use of video lectures, through to an approach where learning can be individualised according to student needs (Lai and Hwang 2016). Pre-prepared lesson materials can range from teacher-produced videos and screencasts to the provision of mathematics resources produced with software such as *GeoGebra* and the use of instructional videos created by others such as *Khan Academy* and *WooTube.* To date, research on the flipped learning approach has predominantly been conducted within the field of tertiary education (Zainuddin and Halili 2016), and studies that interrogate the pedagogical practices that have emerged from flipped learning in secondary mathematics classrooms and the resulting students’ perceptions are limited.

Blended learning approaches such as flipped learning have several benefits that may potentially address some of the significant issues in mathematics education. Firstly, emerging research documents an improvement in student engagement due to the anywhere, anytime affordance of flipped classrooms (Cronhjort et al. 2018; Huang et al. 2018). The approach provides students with greater autonomy in their learning and makes mathematics learning accessible for those who may wish to revise challenging or difficult content (Muir and Geiger 2016). Decreasing the need for whole class explicit teaching within every lesson allows the teacher to work more effectively to address the learning needs of individual students. However, as with most approaches, there are disadvantages to the flipped approach. An early study exploring the introduction of iPads in primary mathematics classrooms found the flipped model did not work well in one Grade 3 classroom due to the level of maturity of the students, their self-regulation, and a mismatch between the mathematics content embedded within the flipped approach and the students’ levels of conceptual understanding (Attard and Curry 2012). A later review of flipped learning approaches by Abeysekera and Dawson (2015) critiqued the approach across all levels of education, claiming a lack of evidence as to its effectiveness. This issue related to the difficulty in tailoring the information to the individual needs of students and the ability of students to take responsibility for their learning outside the classroom. The success of a flipped approach relies on the willingness of students to actively engage with the materials before attending their classes as well as their ability to comprehend the information presented. Having unprepared students may result in unproductive classroom time.

While flipped and blended learning approaches provide students with the opportunity to access mathematics content outside of school, it could be argued that traditional textbooks do the same. However, the affordances of digital technology can promote higher levels of interaction, a more personalised approach and more detailed feedback (Attard and Holmes 2020a, 2020b). Even so, Polly and Casto (2019) caution “In a blended learning environment where students engage with technology, exposure to cognitively demanding tasks is not guaranteed” (p.286). Polly and Casto also claim there are widely held beliefs that teachers of mathematics tend to use technology to focus on low level skills rather than activities that promote higher-order thinking. The success of blended learning approaches is also heavily reliant on the teacher, his or her pedagogical content knowledge, and the practices and interactions that occur during classroom time, with or without technology. Similarly, the teacher’s affinity with digital technology and their technology-infused practices are a critical influence on whether digital technology does improve access, engagement and learning in mathematics (Attard 2018).

### Teachers and technology

The role and expectations of the teacher continue to change as technologies continue to evolve (Armstrong 2014). The NMC/CoSN Horizon Report (Freeman et al. 2017) expresses concern over the challenges that have resulted from technological developments:

Teachers now address social and emotional factors affecting student learning, mentor students, model responsible global citizenship, and motivate students to adopt lifelong learning habits. These evolving expectations are changing the ways teachers engage in their continuing professional development, much of which involves collaboration with other educators and the use of new digital tools and resources (p.30).

Freeman et al. (2017) claim that appropriate teacher training, ongoing professional development, research about student learning, and teacher collaboration are critical for the improvement of teacher practice in this digital age. Livingstone (2012) also suggests that digital learning tools require us to rethink “the relations between pedagogy and society, teacher and pupil, knowledge and participation” (p.20). These concerns are reflected in the calls of mathematics education researchers who have found that teachers particularly struggle when using technology in the mathematics classroom when compared to other subject areas (Attard 2015; OECD 2016; Wachira and Keengwe 2011). Adding to the argument for continued teacher support, Hoyles (2018) emphasises the critical relationship between the digital tools with which mathematical knowledge is expressed and mathematics pedagogy. Hoyles also proposes considers the roles that digital tools play in transforming the practices of learners and teachers. She proposes six categories that distinguish the range of potential offered by digital tools in mathematics classrooms:

1. Dynamic and graphical tools;
2. Tools that outsource processing power;
3. Tools that offer new representational infrastructures for mathematics;
4. Tools that help to bridge the gap between school mathematics and the students’ world;
5. Tools that exploit high-bandwidth connectivity to support mathematics learning; and
6. Tools that offer intelligent support for the teacher when their students engage in exploratory learning with digital technologies.

The six categories proposed by Hoyles (2018) are not “mutually exclusive or exhaustive” (p.212). In this paper, the categories were a useful lens to explore the practices observed in this study.

The diversity in schools, classrooms, students, teachers and cultures means there can be no perfect solution for how teachers and students should use technology. Deciding what technology is best for specific students and cohorts and how to use it is a continuing challenge. Dolan (2016) posits that “the answer may lie more in understanding the practices inherent in using technology, rather than focusing on specific types of devices or tools” (p.33). Likewise, the answer to effective practices using technology may lie in the specific pedagogical strategies that improve students’ experiences of mathematics, address the common challenges to students relating to relevance and engagement. Hence, this study explores the following research question:

1. What blended learning strategies are applied by teachers considered by their peers to be effective and innovative users of technology within secondary mathematics classrooms?

The following sub-questions inform the research question:

- What are the perceptions of teachers and students regarding the blended approaches?
- What are the perceived benefits or limitations of blended learning?

## Methodology

The qualitative data informing this paper are drawn from a more extensive study on the effective use of technology in mathematics classrooms from pre-school to Year 12 (the final year of schooling in Australia) (Attard and Holmes 2020a; Attard and Holmes 2020b). The study was located within an interpretive, social constructivism worldview within which it is assumed that reality is socially constructed and that there is no single, observable reality (Corbin and Strauss 2008; Creswell 2013; Merriam 2009). Within a social constructivism view, individuals seek an understanding of the world to developing subjective meanings of their experiences. Such meanings are varied and multiple and lead the researchers to seek a complexity of views rather than limiting meanings to a small number of categories or ideas. Within a social constructivism view, the goal of research is to “rely as much as possible on the participants’ views of the situation” (Creswell 2013, p. 24). Such a view sits well with the goal of this paper to investigate technology-mediated practices from teachers’ and students’ perspectives, acknowledging that meanings are negotiated socially through interactions with others and through the historical and cultural norms that operate within the lives of students and their teachers (Creswell 2013).

Ten case studies were conducted within nine Australian schools. Each case consisted of a classroom teacher, one member of the school leadership team, and a focus group of five to six students. The case studies were conducted in a combination of pre-school, primary, and secondary schools from a mixture of public, private, and Catholic systems in a range of socio-economic and geographic areas. Initial analysis of the data indicated the four participating secondary school teachers all used variations of blended learning approaches which appeared to have a positive impact on the way their students experienced mathematics education. In order to explore this further and gain a deeper understanding of the blended approaches adopted by these teachers, in this paper, we focus in more depth on the data gathered from the four secondary school case studies.

### Participants

As the purpose of the overarching study was to explore effective technology-infused practices, case study teachers were identified through professional associations, professional teaching networks, and referrals, as teachers who are considered by their peers as effective and innovative users technology within mathematics classrooms. The four secondary teachers came from a range of schools in terms of system, geography, access to digital devices, and school technology-related policy. Students participating in focus groups were selected by their teachers from those who had returned consent forms. Teachers were asked to select a representative sample of students from that pool.

### Site contexts

Information in the following context descriptions is drawn from interviews conducted with the nominated school leaders and the case study teachers. Summary details relating to the participants’ schools are included in Table 1 below:

**Table 1:** Case Study details

Case	*Teacher	Experience	School type	Location	**ICSEA (2019)	Students (2019)	***BYOD
A	Adam	Mid-career	Catholic	Metropolitan	1081	1051	Yes
B	Ben	Early career	Public	Regional	1079	1131	Yes
C	Cameron	Mid-career	Catholic	Remote	1021	584	No
D	David	Mid-career	Private	Metropolitan	1131	1331	Yes

*Pseudonyms

**Index of Community Socio-Educational Advantage (http://www.saasso.asn.au/wp-content/uploads/2012/08/Guide_to_understanding_ICSEA.pdf)

***Bring Your Own Device program

#### Case A: Adam

Adam works at a metropolitan school with a strong technology focus. The students at the school each have either an iPad or a laptop. The school offers a curriculum based on project and problem-based learning across all disciplines. All teachers, regardless of subject, use the *Echo* learning management system (LMS). Adam uses *Echo* to store learning activities that combine other technologies such as *Desmos*, *Geogebra*, *Khan Academy*, and instructional videos from *WooTube* (https://misterwootube.com/). He considers his use of technology a flipped learning approach that provides students with access to previous and current lesson and assessment activities. Adam designs his units of work, to begin with a problem or ‘hook’. He integrates questions, videos, and a range of multimedia that the students access independently during their mathematics lessons. Students work individually but through the use of flexible furniture generally arrange themselves into groups where they can discuss their work and provide peer support.

#### Case B: Ben

Ben works at a regional performing arts high school. The school has a BYOD (Bring Your Own Device) program where all students are required to have a laptop. However, not all families can afford to purchase a laptop, and the school has several laptops to loan to students in this situation. The mathematics faculty uses the *Canvas* LMS and *Microsoft OneNote* as the primary teaching and learning platforms. Teachers take it in turn to set up units of work within *OneNote*, which then forms the basis of their teaching and allows individual teachers to populate the *OneNote* file with their teaching and learning materials. Each lesson is structured into three sections: Revision, Mastery, and Extension. The purpose of this structure is to provide differentiated opportunities for all students to achieve a level of success. Students in Ben’s class work from the questions housed in *OneNote*; however, much of their active work is conducted using a strategy of vertical whiteboarding (Forrester et al. 2017) where students work collaboratively on traditional whiteboards that are hung along the perimeter of the classroom.

#### Case C: Cameron

Cameron’s remote school has less of a technology focus than the other three case study schools. The remote location results in difficulty accessing the internet. At the time of the research, the school provided some access through the provision of several laptops. Cameron’s classroom is equipped with an interactive whiteboard (IWB). Cameron’s expertise is in the creative use of graphics calculators, which he uses extensively along with laptops and iPads when available. Cameron also occasionally uses video clips produced by others in his teaching. Although his school does not run a BYOD program, Cameron uses a flipped approach to engage with his students within and beyond his classroom. For example, on the weekend before our observations, Cameron had created a video and emailed it to his students in preparation for the following lesson.

#### Case D: David

David’s metropolitan school has a strong focus on technology, running a laptop program that requires each student to lease a device, and investing heavily in emerging technologies such as the *Microsoft Hololens* mixed reality platform. The school has a formal technology policy and a dedicated framework for twenty-first century skills that embeds ICT capability. David uses *Microsoft OneNote* as an LMS where he stores all learning material and is able to monitor student progress through a class notebook. Technology features heavily in David’s lessons. He uses self-created videos to demonstrate concepts and as a communication tool to students. He also uses a range of other digital resources including *Geogebra* and *Excel*. David’s lessons do not follow a traditional structure. He introduces each new unit through an introductory activity. Students then follow the program as it is set up in *OneNote* through a self-paced, non-linear approach. Each unit of work is structured to allow students to take different pathways depending on their understanding of the topic. During mathematics lessons, students work at their own pace and any class or group instruction, intervention, or other assistance occurs at the point of need.

### Data collection

In each of the case study sites, data was collected from three participant groups: the case study teacher, a school leader, and students. Data collected from the case study teacher included classroom observations, lesson plans, and teacher interviews. Students participated in a focus group discussion and a nominated school leader participated in an interview. The multiple sources of data aligned with recommendations by Yin (2008) to provide a rich picture of each school context and a detailed analysis of the case study teacher’s classroom practices and student perceptions within each case.

Semi-structured interviews of approximately 30 min duration were chosen as a way of garnering in-depth information from teachers and school leaders in each of the cases. The interviews provided an opportunity for the researchers to explore the teachers’ practices, school policies, and beliefs around the use of technology in mathematics classrooms. The open-ended nature of the interviews allowed each interviewee the opportunity to respond to the same set of prompts to increase the comparability of results while also allowing opportunities to delve deeper into participant responses and match any further questions to individuals and circumstances (Cohen et al. 2018). Focus group discussions used prompts that probed the students’ perceptions of technology use in mathematics. Unstructured classroom observations were conducted to triangulate the interview and focus group data and provide further evidence of how the teachers and students were using technology in practice. Observations provided the researchers with rich data about the interactions between teachers, students, devices, and mathematics content.

### Data analysis

Data drawn from interviews and focus group discussions were audio recorded and transcribed verbatim. Observations were video-recorded. Data analysis was conducted in alignment with the research question posed in this paper using NVivo software. Inductive thematic analysis of data from interviews and focus groups for each individual case study was initially carried out. Field notes and observations were used to support further analysis. A cross-case analysis was then conducted and all relevant data from interviews and focus group discussions from the four case studies were collated to provide collective responses to the research question (Cohen et al. 2018; Saldana 2016).

## Results and discussion

Each of the case study teachers in this research were identified by their peers as effective users of technology, yet due to differences amongst school contexts, access to digital resources, and school policies, there were variations in the ways the affordances of those resources were used. There were also significant similarities in their intentions and philosophies regarding the teaching of mathematics with technology. Students’ perceptions of technology use across the four case studies also yielded strong similarities. All the students participating in focus group discussions held positive attitudes towards the general use of technology in mathematics education.

We now present the results where we weave the data throughout our discussion, organised according to the themes that emerged from the analysis of the data when aligned to the research questions. The themes that did emerge are inter-related, and the order that they are presented in does not necessarily imply a hierarchy of importance. However, we do propose to work from an outside view (devices) to an inside view (practices). We begin with a brief discussion of the variation in access to devices across the cases to provide context for how their affordances were utilised in the application of blended learning strategies.

### Access to devices

Blended learning evolved at each of the case study schools for different reasons that were often influenced by individual teachers, student needs, and access to devices. At school A, blended learning evolved from a mathematics faculty approach to speed up the teaching of mathematics content in the upper secondary classrooms, allowing more time for revision. To date, this has resulted in a dramatic improvement in student learning outcomes at school A. At school B, the blended learning strategies evolved from a need to provide access to learning for students who had been absent from mathematics lessons. In school C, blended learning is slowly being implemented by individual teachers who have an affinity with technology use as more students gain access to devices. In its current form, Cameron uses this approach to communicate with students and provide access to online resources such as videos. At school D, the blended learning model is not implemented school-wide. Rather, it evolved through the case study teacher’s process of experimentation with using *OneNote* and creating instructional videos at a previous school. David achieved success with this approach and decided to continue the practice in his current school. Although David is a Head Teacher, his blended learning strategies are not implemented by others in his faculty.

As described in the methodology section, there were variations of access to devices amongst the schools that impacted the blended learning strategies employed and were influenced by the school system, geographic location, and school policy. Access to devices ranged from high (case D) to minimal (case C). Denise the leader of Teaching and Learning Innovation at school D explained that her school’s philosophy regarding devices is to provide students with all the technology they require without them having to resort to using their own devices (phones). In contrast, Caitlin, Head of Mathematics, explained the challenges faced at the case C school. Although encouraged to bring a personal advice to school, many students fail to do that for a variety of reasons. Caitlin’s school does have a bank of laptops to loan students when this occurs. Challenges accessing the internet may be one factor influencing teacher and student use of devices: “I think we only started putting our toe in the water there and part of that is because of the problems with the NBN (National Broadband Network) and the internet” (Caitlin, leader, case C). An interesting comment from Caitlin implied a tension between a traditional pen and paper approach to mathematics teaching and learning and a technology-based approach:

I also find that in reality, the kids have to use pen and paper for doing a lot of their maths...Microsoft equation editor is very time consuming and very unwieldy, so the laptops aren’t something we use. (Caitlin, leader, case C)

This comment may imply resistance to disrupting traditional practices in mathematics, yet in reality may relate to the lack of devices available, in contrast to a different type of technology integration seen in cases A and D where most of the students completed their work on their devices. In a similar scenario to case C, Ben’s students (case B) predominantly completed their working on traditional whiteboards which they then photographed and uploaded onto their devices. Although access to devices varied across the cases, evidence emerged from the data to indicate that regardless of the level of access, the teachers’ blended learning practices were still able to improve the students’ access to mathematical learning resources as a result of the affordances offered by the available technologies.

### Redefining classroom spaces through blended learning

A feature of blended learning amongst three of the four case studies was the use of learning management systems as repositories for teaching and learning resources including a range of multimedia, and for communication between students and teachers. Evidence gathered from the leaders, teachers, and their students highlighted how the affordances of such systems, combined with affordances offered through embedded multimedia within the LMS, significantly increased students’ access to mathematics resources, including student access to the teacher. Although there were differences in how and to what extent teachers used the affordances of the LMS, there was noteworthy alignment in the intentions of school leaders and teachers for using an LMS for mathematics teaching. The intentions spanned from providing convenience for students and teachers by providing a central place for all course material, to the provision of differentiated tasks. Andrew, Head Teacher, Mathematics (case A) gave this explanation: “Because we want our kids to have the apps there, to access the work through our learning management systems or go onto *Desmos* or *GeoGebra* and graphically see what’s going on with the mathematics”. Belinda, Head Teacher, Mathematics (case B), listed additional affordances that were beneficial for teachers and students in their use of a combination of two LMS (*Canvas* and *Microsoft OneNote*) including using them to ensure students know where to find their set work. Similarly, Andrew (case A) outlined how the use of an LMS provides access to learning materials, enabling a blended learning approach to occur:

Technology really helps us because we use a platform called *Echo* and teachers can post videos on there, put all our homework onto there. Without those platforms it would be hard for students to actually go home and do what they did in class. If they don’t understand something they can go home and redo it. Without *Echo* and the technology that won’t be able to happen.

The teachers’ intentions regarding the use of an LMS were more focused on the affordances relating to student learning rather than logistics. Ben (case B) talked about the flexibility it offered his students, allowing them to move between their work, saying: “students have the flexibility to move between which area they are working in, they are not constrained by what is up on the board” (Ben, case B). This strategy builds on the more traditional use of blended learning approaches as defined in the literature (Gilboy et al. 2015), by promoting non-linear learning opportunities. In a school where absences are frequent due to extra-curricular activities, the ability for students to catch up on work was considered a benefit. This affordance is significant in mathematics classrooms due to the hierarchical nature of the subject and the misconceptions that can occur if foundational concepts are not understood (Boaler 2009). Ben also discussed the time saved from having class notes in electronic form, explaining in the following quote:

It is a combination of them having a record of everything that I have written down, and when they miss class, they have got a record of what we did on that day. And the parents often contact me and say can you let me know what you did, and I say: Just get your child to open One Note and show you, and it is all there. (Ben, case B)

Comments from the students aligned with the teachers’ intentions for using an LMS:

“Yeah so what we do is we go into *Canvas*, and that is basically where all of our subjects are. And from there you can go into your mathematics section and then get on to *One Note* that way” (Student, case B). A high level of organisation of content and activities appeared to be important to these students, indicating a desire for transparency on the part of the teachers. These findings extend Hoyles (2018) category of digital technology use, *tools that offer connections between school mathematics and learners’ agendas and culture* through the elimination of physical classroom and timetable-imposed boundaries. The following quote highlights a significant benefit of LMS to provide students with continual access to learning beyond the classroom walls:

We can go to *Echo* and see what topics we have in the future and while the teacher’s teaching we can go through and see if we understand everything and going ahead and other students can learn what the teacher’s teaching. That’s what’s different. (Student, case A)

The above quote indicates the value of allowing students some flexibility and opportunities to prepare for upcoming lessons as well as the chance to look back and revise prior learning, providing another example of how a blended learning approach can be extended and made multidirectional. The accessibility of past and present learning resources also appears to promote the building of connections across mathematical content, evidenced in this quote:

No, it’s not just the videos. I kind of like how the whole - because we work on OneNote as well and I like how that’s all displayed, and it’s just very organised, it’s very section by section doing this, that links in with that topic, this links in with this topic. It just connects, just everything is all connected. (Student, case D)

In addition to improving connections within mathematics, the use of an LMS has built connections within faculties (case B), between school and families (case B), and between teachers and their students. The boundaries between students’ lives outside schools and the four walls of the mathematics classrooms have blurred (all four cases): “It’s not uncommon for me to get an email at any given time from our kids here because they are looking forward to going to mathematics and want to learn more” (Andrew, leader, case A). Evidence of increased access to mathematics content is now explored. These findings expand the category of *tools that exploit high-bandwidth connectivity to support mathematical learning* proposed by Hoyles (2018), who discusses connectivity in relation to having students collaborate on online mathematics tasks. The connectivity observed in this study is pedagogical in nature and highlights how each individual teacher’s nuanced digital practices influence how students experience mathematics education.

### Changes in access to mathematics content

The methods of technology use across the four case studies resulted in improvements in collaboration and student engagement and improved opportunities to differentiate learning. They also provided the students with access to new or different representations of concepts through various multimedia in alignment with Hoyles (2018) category *dynamic and graphical tools* that provide opportunities for mathematics to be explored in diverse ways. An affordance of the technology use within the four case studies was, in some cases, the ability to improve access to mathematical concepts and representations through tools such as *GeoGebra* and *Desmos*. The following quote from Adam (case A) provides insight into his experiences in using technology to increase student access to mathematics:

But if it’s a tool that the students can also engage with, well then that just becomes more powerful. Because then students - when students can actually interact with mathematics, where they can do something, and something comes back to them, then there’s a lot more meaning. Then when they can use that to then demonstrate to another student. I mean, that’s kind of the epitome of where you want the student’s learning to go. When they can teach something to someone else. If they can do that, well then you know you’ve succeeded. (Adam, case A)

The case study teachers discussed the ways technology assisted in promoting visualisation of mathematical concepts through the use of various apps such as *GeoGebra* and *Desmos*. However, David (case D), cautioned that he does not use technology unless it the best option for representing concepts:

I guess you’ve got to know what the limitations of your tools are and what your tools can do. Then you’ve got to say well, once you’re that comfortable with them you could say oh, okay, usually I would just draw it on the board but is there a better way to represent those things? (David, case D)

David goes on to provide the following discussion of mathematics topics that lend themselves well to technology use:

If you’re going to teach probability then it probably makes sense to be able to use something like Excel, or even like some rudimentary program in Python or something like that, to be able to show okay, this is what happens if I flip a coin 10 times, 100 times, 1000 times, 10,000 times. If you’re going to do something like functions transformations and you’re not opening up something like GeoGebra or Desmos or something like that, then I’m not sure what you’re doing. (David, case D)

Cameron spoke about the dynamic nature of digital representations when compared to using static objects and the benefits of being able to manipulate representations to improve students’ conceptual understanding. The affordance of alternative mathematical representations was something that students also found to be of benefit, aligning with the teachers’ intentions. This quote is typical of the groups’ sentiments:

I think it’s also good that it kind of allows for all sorts of learning because I’m not really visual. But like, yeah, the GeoGebra and the YouTube and stuff is kind of visual and kinaesthetic with all the stuff in there. It allows for a bit of everything. (Student, case D)

### Affordances promoting access through differentiation

Three out of the four case study teachers indicated an explicit desire to differentiate learning for their students. Ben (case B) was in his first year of teaching when he began to provide differentiation through technology. As a beginning teacher, he noticed a diversity in student abilities even within secondary classes that were streamed. This led to the use of *OneNote* and the structure of offering tasks at three different levels within each lesson that has been adopted across the mathematics faculty at his school. Providing differentiated tasks via technology rather than more traditional modes has been beneficial for Ben’s students, as he describes below:

The technology allows students to seamlessly move between them [the tasks] and also, surreptitiously move between it. So, they are not announcing to the class they are one level or another, they can just work on what they are comfortable with. And that can work both ways, especially with the girls, who are sometimes hesitant to show that they understand it and they are smart – because often you find that with girls, they don’t want to stand out. (Student, case B)

The removal of stigmas associated with either being ‘too clever’ or ‘not smart enough’ has significant potential implications for mathematics students, influencing self-efficacy, engagement, and beliefs about mathematics (Boaler 2015). These affective issues have been found to influence the decisions of students to continue or discontinue the study of mathematics beyond the compulsory years.

Although his intentions are similar to Ben’s, David’s design of differentiated learning through technology is more complex. Rather than designing three distinct levels of tasks, David designs a broad range of tasks that allow his students to progress through the work in different ways through varied trajectories. All of his students begin at the same point, and he intends that by the end of a unit, they have all achieved the set outcome. The combination of differentiated tasks and having a range of learning resources available through OneNote results in David having the time to monitor student progress and provide assistance and intervention when required:

I’ll be walking around the classroom, and I’ll discover a misconception in one student, and I will correct that. Then I’ll discover a misconception in a second student, and I know okay, that’s on me. Now we can all come to the front and I can say okay, in the video I said this and I reckon I didn’t explain that very well. Or, but - or [in the video] I said this and but there’s a bit more to it or whatever it might be. (David, case D)

This level of monitoring also allows David to provide ‘just in time’ support to small groups through microteaching, which appears to have increased his students’ confidence in asking for help. During the classroom observations, all four teachers were observed to be conducting microteaching episodes and providing support to individual or small groups of students as the need arose. David’s students indicated awareness and appreciation of the differentiated learning provided by him. This comment exemplifies the group’s feelings:

Mr [David] came around and noticed that I was still watching the same thing and it was just I wasn’t quite understanding why he was doing one of these steps. It was just like why is that the next thing to do? Not after he explained it to me, I sort of got better and then I just put it into practice and got better at it. (Student, case D)

Similarly, the students at Case A realised how technology provides differentiation:

Without technology I would feel less motivated to do maths because instead of - we have step-by-step guides that we use and interactive activities on the technology. If I needed help with maths then I’d rely on technology the most but if I didn’t have the technology I would struggle a lot and I’d lose motivation to continue doing my homework. (Student, case A)

The affordance of technology that provides opportunities for students to work at their own pace and engage with tasks that are suited to their academic abilities was a significant theme that emerged from the data. The combination of affordances accessed by the teachers resulted in more complex forms of blended learning that may not necessarily have been intended. Instead, in some cases, blended learning evolved as an unintended but valuable outcome of a combination of technology use and effective practice.

### The use of videos in blended learning

Video use emerged as an element of technology use that has a significant impact on students’ learning experiences in mathematics. The intention of teachers for using the videos includes providing more time for interaction during class, in alignment with the literature on flipped learning approaches (Gilboy et al. 2015; Lo and Hew 2017). Teachers also indicated that the use of videos (self-created or sourced from YouTube) is more engaging for students than a traditional textbook.

The use of videos was one of the most prominent features emerging from the student focus group discussions at each of the schools. Students reported the videos to be more stimulating than traditional texts, and they appreciated the ability to re-watch the videos and found that videos sourced from *YouTube* added a different perspective on the mathematics content. Although it was clear the students at all schools found the use of videos an important benefit of technology, one student did comment that they could be distracting.

In David’s classroom (case D), videos were used innovatively to provide feedback to students, creating a bidirectional model of flipped learning. David prefers to respond to students’ work through individual videos that allow him to communicate with students outside school hours in a highly personalised manner. He explains his motivations in this quote:

If the student sort of hands me in a diagnostic test and I can set up a camera and I can bring in their diagnostic test and say hey, I’m just going to go through your maths work here in the next two or three minutes. I’m giving them some really, really specific feedback on what they’ve done. (David, case D)

David’s intent regarding the video feedback and his more general use of self-produced videos had strong alignment with the perceptions of his students. This quote is typical of the group’s sentiments:

This way of teaching just gets to me, especially I'm a visual, so the videos do really help. (Student, case D)

### Self-paced learning

Another dominant theme that emerged from the research was the element of self-paced learning that was afforded by the use of blended learning strategies. Within three of the case studies, the explicit intentions of the teachers were to provide differentiation and all four teachers sought to provide access to learning beyond the limitations of school timetabling. Although the term ‘self-pacing’ was not featured in the teacher interviews, it was a noticeable feature in the student focus group discussions. The students indicated they valued the opportunities to either re-visit prior learning or move forward with extension activities. The following quote is representative of the perceptions of many students:

I think just the different speeds of the students in the class and some students may take longer to learn one topic and some students may take - they might get the topic quick. Going back to the other questions, that’s why technology’s there. We can go to *Echo* and see what topics we have in the future and while the teacher’s teaching we can go through and see if we understand everything and going ahead and other students can learn what the teacher’s teaching. That’s what’s different. (Student, case A)

Students who struggle with understanding particular concepts also demonstrated strong support for self -paced learning: “...if you’re not having a good day and whatever you can watch the same video until you understand it or keep moving on without knowing that you’re going to miss something important.” (Student, case A)

Similarly, students who are confident and like to move ahead with their learning expressed an appreciation for self-paced learning:

So for me, I kind of - I’m one who loves to do heaps and heaps of questions. I think that’s the way I learn, by doing lots of stuff, and the same sort of thing as Adam; in other classes where you’re going at the same pace as everyone else I always found that I would be done with the questions. (Student, case D)

The use of self-paced learning afforded by technology requires discipline on the part of the students. Evidence from this research suggests the students involved were engaged in their learning and were able to remain on task while working independently. The following quote provides a testament to David and his effective use of technology:

... it gives me the sense of the trust between him as a teacher and us as a student, the trust that we’re working and we’re on task and it then is up to us to stay up and to do what you’re meant to be doing and to stay on topic and to make sure you’re up with the rest of the class and not way behind. (Student, case D)

## Conclusion

This goal of this paper was to explore the blended learning strategies applied by four mathematics teachers considered by their peers as effective and innovative users of technology. To do this, we interrogated data drawn from classroom observations, interviews with the teachers and nominated school leaders, and student focus groups. We explored the teachers’ and students’ perceptions of blended approaches and the perceived benefits or limitations within mathematics education. The results of this study provide some illustration of the potential benefits of blended learning strategies to begin to break down classroom walls and redefine learning spaces, removing barriers between home and school and making learning more accessible in a multiple of ways. The variation in blended and flipped learning approaches evidenced in this study aligns with the claims of Wasserman et al. (2017). They claim that flipping learning has different meanings for different teachers, with little consistency in models. For example, the most basic use of an LMS in this study appeared to extend blended learning approaches, shifting from unidirectional to multidirectional learning, and increasing opportunities for teacher-student interactions.

Regardless of the level of access to devices across the case studies, each teacher featured in this paper was able to use technology to improve their students’ access to mathematics learning resources. The use of an LMS provided opportunities for teachers to accumulate a range of digital resources that were organised and made accessible for students, providing them with access to alternative mathematical representations, alternative teaching methods made accessible beyond the school timetabled lessons. The technology provided opportunities to redefine learning spaces and teaching practice. In addition, technology promoted connections between teachers and students; between teachers and teachers; and between teachers, students, and families. Technology use promoted self-confidence and increased self-efficacy due to the differentiation afforded by the LMS. This then allowed the teachers the ability to provide ‘just in time’ support in combination with student self-paced learning opportunities. These students no longer view mathematics as something that only belongs in the classroom.

There was also evidence that the teachers did find technology effective for promoting mathematical thinking and communication. The affordances of technology that allow mathematics concepts to be represented in multiple ways, particularly those that are visual and dynamic, clearly assisted student understanding and their capacity to make connections between mathematics concepts. These connections were supported through LMS use, providing students with multiple self-paced pathways through the mathematical content, allowing them to see the ‘big picture’, because they were not restricted to the content being presented by the teacher on any particular day, as would be the case in a more traditional classroom setting. In relation to mathematical communication, three of the four case study teachers made use of their LMS to provide an active means of communicating with students about mathematical work. The ability to offer easily accessible, timely and personalised feedback through the LMS was recognised by these teachers as feature to extend mathematical conversations with students and to focus on developing their mathematical thinking,

While the results of this small study were positive, caution must be exercised. The teachers featured in this paper were identified by their peers as effective and innovative users of technology. They displayed an affinity with technology and a commitment to its use in mathematics education that may not be representative of the general teaching population. Their implementation of blended learning often resulted in an increased workload. This workload included activities such as developing and curating digital resources and communicating with students outside the classroom timetabled hours. As mentioned at the start of this paper, this increased workload will have been experienced by all teachers during the COVID-19 crisis, regardless of their affinity with and attitudes towards the use of technology in their mathematics classrooms. We argue that a deeper understanding of the nuanced approaches to blended learning and teachers’ and students’ perceptions regarding their experiences will assist in more effective implementation of strategies to improve the mathematics education experience of young people, regardless of when and where the teaching and learning occurs. The provision of professional development that equips teachers to develop effective teacher/student communication techniques required for online and blended learning should be a priority. In a post pandemic world, a large proportion of teachers will be well-positioned to take advantage of new skills gained during the crisis, increasing the likelihood that blended learning will become more widespread in the future. The strategies used by the teachers in this study reveal a variety of pedagogical pathways through which blended learning can be adapted for improved student learning.

Although the uses of technology highlighted in this study may not appear to be particularly innovative, this paper does contribute to existing literature to demonstrate how mathematics teaching practice and student learning has the potential to be enhanced and redefined through the use blended learning strategies and sound practice, as evidenced in the alignment between the teachers’ intentions and the experiences and perceptions of their students. Schools do not need to make significant investments in complex technological devices and expensive software to augment their practices and provide individual and flexible learning opportunities that reach beyond mathematics classrooms. Although the data presented in this paper is limited to four case studies, this did allow the researchers to place each of the cases under a microscope to investigate the blended learning practices that occur across individual and unique school contexts. Findings from this paper may promote further and deeper investigation of how what appear to be small changes to technology use can lead to improvements in the student experience of mathematics, potentially influencing students’ choices to continue the study of mathematics beyond the compulsory years.

The themes emerging from our analysis point to several elements as key considerations for the use of technology in teaching and learning mathematics. Firstly, the provision of devices for students raises questions which are highly dependent on each school context and the resources and technical support available. Secondly, the choice of an LMS and the ways in which it is employed are crucial if affordances enabling blended learning approaches are to be realised. Next, the affordances of mathematics specific tools such as *Geogebra*, *Desmos*, and mathematics videos should be appraised for their capacity to illuminate mathematics concept from multiple perspectives. Finally, consideration should be given to how technology can allow for differentiation of mathematics content, pathways, and the pace at which students make progress as they learn. The teachers in this study demonstrated how these considerations were central to their pedagogical decision-making, providing a sound basis for the judicious use of technology for enhancing their students’ learning in mathematics.
